# The Effect of Resin Type and Placement Technique on the Hardness of Resin-Based Composites Polymerized with LED and UV Light-Curing Units

**DOI:** 10.3390/polym17060774

**Published:** 2025-03-14

**Authors:** Ayse Nurcan Duman, Arife Dogan

**Affiliations:** Department of Prosthodontics, Faculty of Dentistry, Gazi University, 06490 Ankara, Türkiye; adogan1956@yahoo.com

**Keywords:** bulk, hardness, incremental, light-emitting diodes, resin-based composite, standard, ultraviolet

## Abstract

The aim of this in vitro study is to evaluate the effect of resin type and placement technique on the hardness of resin-based composites (RBCs). A total of 300 samples consisting of five RBCs (Filtek Z250 microhybrid, Filtek P60 packable, Tetric Ceram hybrid, Admira ORMOCER, and Tetric Flow flowable RBCs) were prepared. Each RBC was placed into Teflon molds with a 4 mm diameter and 2 or 8 mm depths with standard, bulk and incremental techniques and was polymerized by second-generation LED (Hilux Ledmax 1055, 229.153 mW/cm^2^) and UV (ELC-410, 26.106 mW/cm^2^) light-curing units (LCUs) in standard mode (n = 10). The Vickers hardness number (VHN) was measured from the top and bottom surfaces of the RBCs. Data were statistically analyzed with a one-way ANOVA. Multiple comparisons were made using the Tukey, Scheffe, and *t*-tests (*p* < 0.05). The VHN of the RBCs polymerized with LED and UV LCUs varied between 110.33 and 25.16 and between 104.86 and 34.20, respectively. The Tetric Flow RBC did not polymerize with the LCUs on either surface. The RBCs placed using the bulk technique could not polymerize with the UV LCU on the top surface, except for the Filtek P60 RBC, but showed a higher VHN on the bottom surface. These significant findings highlight that the hardness is specific to the RBC material and placement technique.

## 1. Introduction

Light-cured resin-based composites (RBCs) are restorative materials widely used in direct filling or indirect restorations such as inlays, onlays, crowns, endodontic posts, and cores [1,2,3]. Hybrid RBCs are preferred by clinicians as universal restorative materials for both anterior and posterior restorations due to their superior aesthetics, strength, and durability [4,5]. Packable RBCs are characterized by a high filler load and a filler distribution that provides a stiffer consistency compared to hybrid composites. ORMOCER combines glass-like filler (inorganic) and polymer (organic) components with different technologies and is recommended for stress-bearing posterior restorations. Flowable RBCs are low-viscosity RBCs produced by maintaining the same small particle sizes of traditional hybrid RBCs but reducing the filler content [4,5,6].

It is advised to place light-polymerized RBCs using the conventional standard technique at a maximum curing depth of 1.5–2 mm. When the cavity preparation exceeds a 2 mm depth, especially class II restorations, the incremental layering technique based on the oblique or horizontal placement of 2 mm thick RBC layers is recommended to reduce polymerization shrinkage and obtain adequate mechanical properties [6,7,8,9,10]. However, it takes a long time, and if not properly placed, void formation or moisture contamination may occur among layers and at the margins of the restoration, leading to the weakening of the restoration or microleakage [9]. Bulk-filling techniques and bulk-fill RBCs have been developed to overcome these problems and simplify the RBC placement technique in deep cavities [11,12]. Manufacturers claim that bulk-fill RBCs can be placed using the bulk technique in one single step up to 4 mm or even 6 mm increments and adequately polymerized [7,10,12,13].

The polymerization of light-activated RBCs occurs through the photoinitiators contained in their composition [14]. In the 1970s, the first light-curing RBCs commonly used benzoin methyl ether photoinitiators and were polymerized with ultraviolet (UV) LCUs with a wavelength of approximately 366 nm [10,15]. Although clinical procedures were facilitated by UV irradiation, it was reported that UV light might cause skin cancer, eye lens damage, and mutagenic effects, and resin polymerization could not be completed due to the limited light penetration depth [15,16,17,18,19]. Considering the potential adverse biological effects, UV light was replaced by visible light sources [16,17,18,19]. The original UV-curable RBCs were reformulated, and the combination of camphoroquinone (CQ) and amine was added to the formulation because most RBCs employ CQ as a photoinitiator, which has an absorption range of 370–500 nm in the visible light spectrum, with a peak absorption at 468 nm [20,21].

Currently, four types of visible light sources are available: argon-ion lasers, plasma arc curing (PAC) lamps, quartz–tungsten–halogen (QTH) units, and light-emitting diode (LED) LCUs [22,23]. However, solid-state LED LCUs are the most preferred LCUs for polymerizing RBCs. Studies have reported that RBCs polymerized with second-generation LED LCUs have a high degree of polymerization, a stable three-dimensional structure, and a great curing depth [24,25,26]. And the irradiation time can be reduced without causing a significant loss in mechanical properties [24,25]. However, some manufacturers use less CQ to achieve a whiter or more translucent color in RBCs due to the intense yellow color of CQ, which can be a problem when polymerizing light RBCs with narrow-spectrum second-generation LEDs. Since it has been reported that second-generation LED LCUs do not properly activate RBCs containing co-initiators that absorb at shorter wavelengths in addition to CQ (<410 nm), third-generation LED LCUs (polywave) have been developed for curing RBCs containing other photoinitiators in addition to CQ, which are a combination of blue and ultraviolet LEDs and have a broader spectral range and greater output than second-generation LEDs (single wavelength) [27,28]. However, the studies showed that third-generation LEDs do not improve the hardness at the bottom surface of the RBCs [26,29,30]. Since second-generation LEDs do not produce heat, and the most common photoinitiator used in RBCs is CQ, second-generation LEDs are currently routinely preferred over third-generation LEDs to avoid possible gingival or pulpal irritation [26,31].

Despite advances in RBC and LCU technology, insufficient polymerization is a major problem. It causes an increased local tissue reaction, microleakages, recurrent caries, and pulp irritation due to a residual monomer content, which all negatively affect the physical, chemical, and mechanical properties of RBCs, such as surface hardness, dimensional stability, wear resistance, water absorption, color change, and biocompatibility, leading to restoration failure [10,13,32]. The performance of biomaterials is most frequently evaluated using laboratory tests [33]. Hardness is a widely used indirect test to evaluate the efficiency of polymerization of dental materials [28,34]. The term “hardness” refers to the resistance of a material’s surface to penetration and its ability to resist scratches and abrasion [28]. The Vickers test is a non-destructive method and the one most commonly used to determine the hardness of harder and brittle materials [5,35].

Although there are several studies evaluating the hardness of RBCs, most studies focus on high-intensity curing for rapid polymerization [5,6,7,11,24,26,27,28,29,30,35]. However, especially in deep cavities and large restorations, the polymerization of more RBC layers with high-light-intensity LCUs is a major disadvantage, as it leads to increased microleakages and the decreased mechanical properties of the RBC material, such as elastic modulus and hardness, due to low polymeric chain formation and shrinkage stresses, and irreversible pulp damage due to excessive heat in the tooth [22,23,24]. If the photoinitiator does not absorb the emitted light, the higher energy output of the LCU will not improve the polymerization of the RBC [17,36]. However, there are no recent studies on polymerized RBCs with low-intensity LCUs such as second-generation LED and UV LCUs. The most important advantage of second-generation LED and UV LCUs is their lower energy consumption. Since they produce less heat while hardening the resin, they do not cause a temperature increase or pulp sensitivity in the tooth. UV-A rays in the range of 320–400 nm are generally used in dentistry, which are less damaging to the body. The other advantage is their efficiency in curing the monomer with a high level of conversion in the surface layers due to their ability to prevent oxygen-inhibited layer formation on the surface [15,17]. Another advantage is their ability to prevent color changes in previously cured or curing material [17].

The purpose of this in vitro study was to evaluate the effectiveness of resin type and placement technique on the Vickers hardness of different RBCs (microhybrid, packable, hybrid, ORMOCER, and flowable RBCs) placed using standard, bulk, and incremental techniques and polymerized by LED and UV LCUs. The null hypothesis is that both the RBC types and placement techniques tested would be affected by the microhardness of RBCs polymerized by LED and UV LCUs.

## 2. Materials and Methods

### 2.1. RBC Materials

Five different light-cured RBCs (microhybrid (Filtek Z250, 3M ESPE, St. Paul, MN, USA), packable (Filtek P60, 3M ESPE, St. Paul, MN, USA), hybrid (Tetric Ceram, Ivoclar Vivadent, Schaan, Liechtenstein), ORMOCER-based (Admira, Voco GmbH, Cuxhaven, Germany) and flowable (Tetric Flow, Ivoclar Vivadent, Schaan, Liechtenstein) were used in this in vitro study. RBC materials used in this study and their compositions are presented in Table 1.

### 2.2. Light-Curing Unit and Assessment of Power Output, Emission Spectra, and the Distribution of Irradiance as a Function of Time

Before the experiments, the light intensity, power output, and emission spectra of LED LCU (Hilux/Ledmax 1055 s-generation LED curing lights, Benlioğlu Dental Inc., Ankara, Türkiye) and UV LCU (ELC-410 light-curing system, Danbury, CT, USA) used in the study as a function of time were measured from the output diameter of 11 mm with 60° bent fiber opticand 11 mm curvature light guide tips using a handmade photoluminescence (PL) system (Department of Physics, METU, Ankara, Türkiye), respectively. In PL experiments, an LCU, whose power is sufficient to obtain PL signals from the sample, is any laser that can provide a beam of photons with a greater amount of energy than the band gap of the studied material. In the experiments, an Nd:YAG (neodium-doped yttrium aluminium garnet (Nd:Y3Al5O12)) laser was used to excite the samples. It was operated with radiation at 532 nm and produced photons with an energy of 2.33 EV. The monochromator spectrally resolves the signals, transmitting a selectable narrow band of wavelengths of light chosen from a wider range of wavelengths coming from the sample. The wavelength range that is transmitted from the monochromator varies with the choice of grating, consisting of a substrate with many parallel grooves on its surface and overcoated with a reflective material, such as aluminum. The monochromator was controlled through the RS232 port of the computer by using programs written in LABVIEW. To detect PL signals, a Hamamatsu C7041 multichannel detector with 1044 × 256 pixel S7031–1008 serial CCD (charge coupling device, Hamamatsu Photonics K.K, Hamamatsu City, Japan) image sensor was used.

**Figure 1:**
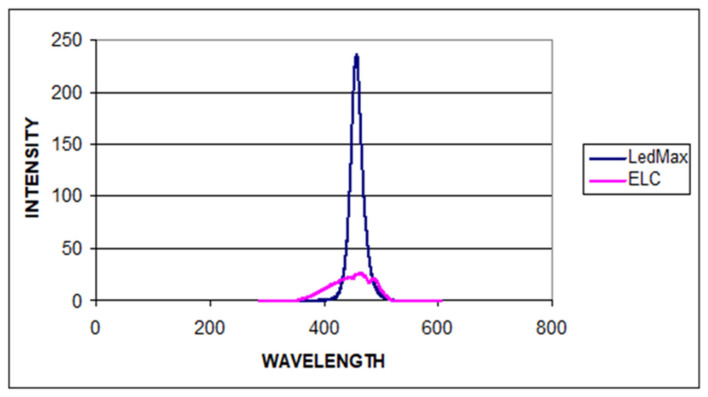
The emission spectrum and the wavelength peaks of LED and UV LCUs assessed using the photoluminescence system.

The emission spectrum and the wavelength peaks of LED and UV LCUs assessed using a photoluminescence system are shown in Figure 1.

The area under the curve in Figure 1 represents the total power output of the spectral flux of LCUs. The spectral flux represents the optical power output from the LCUs in milliwatts (mW) at each given wavelength (nm). The spectral irradiance of the LCUs was determined by dividing the output power by the area of the light exit window or light guide tip (mW/cm^2^) [25].

Figure 1 shows that the spectrum of the LED LCU is effective between the wavelengths of 418.724 nm and 512.268 nm. It peaks at 458.595 nm and 229.153 mW/cm^2^ light intensities and shows a much narrower spectral distribution than UV LCU. However, the spectral output of the UV LCU is effective between the wavelengths of 374.253 and 530.313 nm. It peaks at 465.036 nm and shows a much lower light intensity (26.106 mW/cm^2^), which has a much wider wavelength range than LED LCU. It was observed that the peak of the spectral distributions of LED and UV LCU coincided with the peak of the CQ absorption curve at approximately 460 nm and 465 nm, respectively. Details of the data of the LCUs used in this study are listed in Table 2.

### 2.3. Placement Techniques and Polymerizing of the RBCs

**Figure 2:**
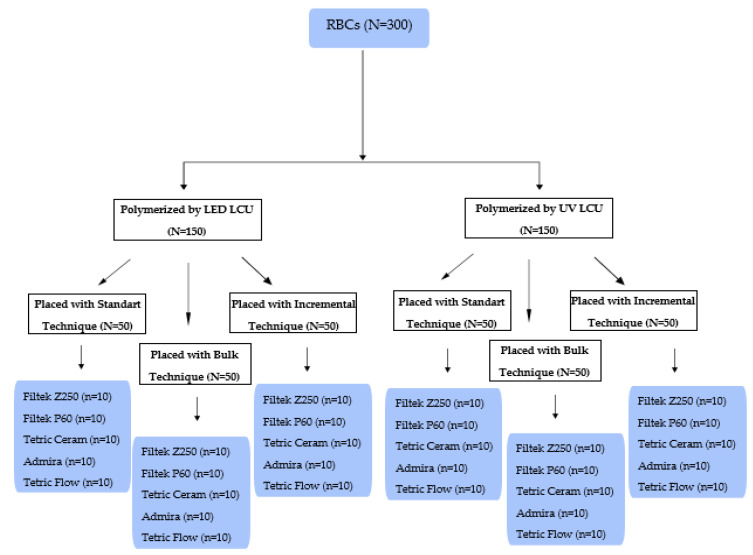
The flow chart of this study.

After assessment of the light intensity of LED LCUs, five different light-cured RBCs (microhybrid, packable, hybrid, ORMOCER-based, and flowable RBCs) were placed into Teflon molds with 4 mm diameter and 2 or 8 mm height using three placement techniques. RBCs were placed into 2 mm depth molds with standard technique as one single 2 mm thickness layer. RBCs were placed into 8 mm depth molds with bulk technique as a single 8 mm thickness layer. RBCs were placed into 8 mm depth molds with incremental technique horizontally as 4 layers with a thickness of 2 mm. The top surface of RBCs was covered with a Mylar transparent acetate strip (GC, Tokyo, Japan) and gently compressed with a 1 mm thick glass slide to achieve a flat test surface and avoid contact with oxygen, which is an inhibitor of polymerization [32]. Then light guide tips were centered on the glass plate, and Mylar strip, and samples were polymerized by LED and UV LCUs in continuous standard mode according to placement technique and curing times recommended by RBC manufacturers. Sixty samples were prepared to polymerize each RBC material by LED and UV LCUs (n = 10). The flow chart of this study is given in Figure 2.

At the end of the irradiation period, glass slides and Mylar acetate strips were discarded, and the samples were carefully removed from the molds. Then, the bottom surfaces of the RBC samples were marked with a pencil, and RBC samples were stored in a dry and dark environment at 37 ± 1 °C for 24 h post-irradiation until testing.

### 2.4. Determining the VHN

After 1 day, the top and bottom surfaces of RBC materials were subjected to Vickers hardness test. In the Vickers testing device, the scratching diamond tip is a pyramid with a square cross-section with an intersurface angle of 136°. To make a hardness image, the measured surface must be parallel to the table of the testing device, and therefore, the surface tested must be smooth and polished well [35]. Since the RBC samples were polymerized on transparent Mylar strips and glass plates, smooth and shiny surfaces were obtained on the top surfaces; they were not subjected to additional polishing. But the bottom surfaces of the RBC samples were polished before the VHN measurements, using a sequence of 600–800–1200-grit silicon carbide papers and Sof-Lex discs (3M ESPE, St. Paul, MN, USA) to obtain a smooth surface. Following this process, the measuring indenter, the Vickers pyramid, was pressed to the RBC samples using a load of 1.961 N to the top and bottom surfaces for 15 s using the Vickers microhardness tester (Shimadzu Version 1.29, HMV-2, Kyoto, Japan).

The two diagonals of the diamond-shaped rectangular trace extending diagonally from one edge to another on the RBC samples were measured digitally with an optical microscope under ×500 magnification (±0.5 µm accuracy). Three tracks were created on each surface approximately 1 mm apart. A total of 1800 measurements were made from the top and bottom surfaces of RBCs tested. Vickers hardness was determined by taking the average of three indentation values for each surface. Mean Vickers hardness number (VHN) was calculated using the following formula [37]:

$$\mathrm{VHN}\frac{2F\sin\frac{136^{\circ }}{2}}{{\left(d1+d2\right)}^{2}}=1.854\times \frac{F}{d^{2}}$$

Here, VHN = Vickers hardness number, F = applied force (kgf), d1 and d2 = the surface areas of the scratch, and d = arithmetic average of two diagonals d1 and d2 (mm^2^).

**Figure 3:**
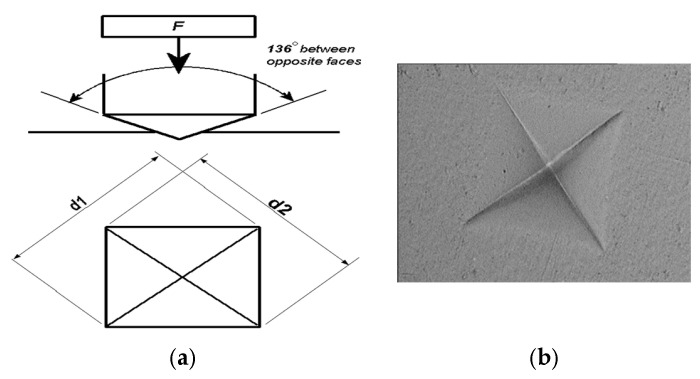
(**a**) Cross section of Vickers pyramid [37]; (**b**) The trace created by a Vickers diamond pyramid on the bottom surface of Filtek Z250 sample used in this study.

The cross-section of Vickers pyramid and the trace created by a Vickers diamond pyramid in Filtek Z250 sample is given in Figure 3a,b [37].

### 2.5. Statistical Analysis

The mean VHNs and standard deviations of the top and bottom surfaces of RBCs tested were analyzed using an SPSS statistical software package program (SPSS 11.5 for Windows, release 6.1.2; SPSS Inc., Chicago, IL, USA). One-way analysis of variance (ANOVA) was applied for the top and the bottom surfaces to test whether there was a significant difference in VHNs. Because of the significant differences found statistically in the VHNs with respect to placement techniques, multiple comparisons were performed using the Tukey HSD post-hoc test to identify pairwise comparisons at a significance level of 0.05, following ANOVA.

Additionally, since significant differences (*p* < 0.05) were found statistically in VHN with respect to RBC materials (*p* < 0.05), multiple comparisons were performed using Scheffe and *t*-tests at the *p* < 0.05 and *p* < 0.01 significance levels to determine which groups these differences originated from.

## 3. Results

According to the one-way ANOVA, the mean VHNs and standard deviations of the RBCs polymerized with the LED LCU on the top and bottom surface are given in Table 3.

**Figure 4:**
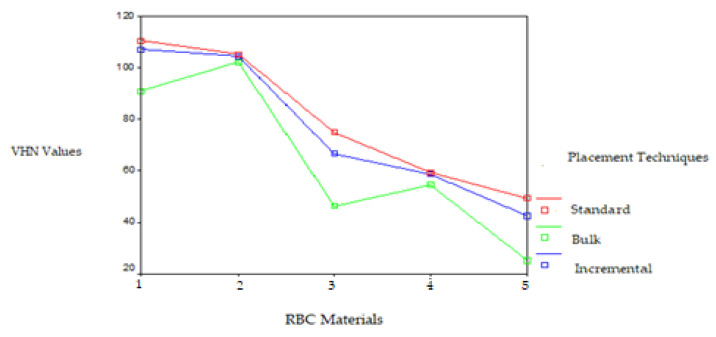
VHNs of the RBCs polymerized with LED LCU on the top surface. RBC materials: 1. Filtek Z250 microhybrid, 2. Filtek P60 packable, 3. Tetric Ceram hybrid, 4. Admira ORMOCER. and 5. Tetric Flow flowable RBCs.

**Figure 5:**
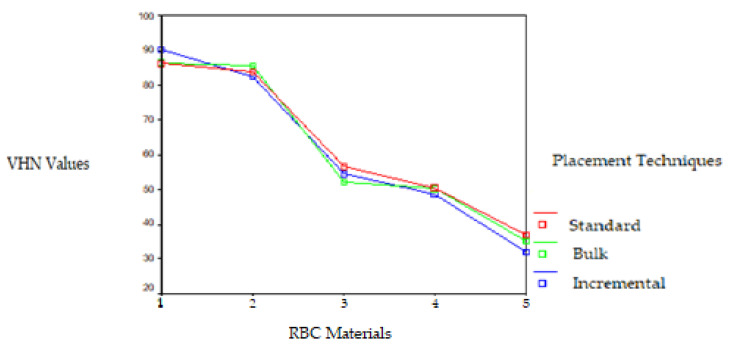
VHNs of the RBCs polymerized with LED LCU on the bottom surface. RBC materials: 1. Filtek Z250 microhybrid, 2. Filtek P60 packable, 3. Tetric Ceram hybrid, 4. Admira ORMOCER, and 5. Tetric Flow flowable RBCs.

Significant differences were observed in the VHN of the RBCs polymerized by the LED LCU on the top and bottom surfaces. The VHNs of the RBCs polymerized by the LED LCU ranged from 110.33 to 25.16. While the Filtek Z250 RBC placed using the standard technique showed the highest VHN and the Tetric Flow RBC placed using the bulk technique showed the lowest VHN on the top surface, the Filtek Z250 RBC placed using the incremental technique showed the highest VHN and the Tetric Flow RBC placed using the incremental technique showed the lowest VHN on the bottom surface. The Filtek Z250 microhybrid and Filtek P60 packable RBCs placed using the three techniques and polymerized with the LED LCU resulted in a higher VHN on both surfaces than the other RBCs. This difference was found to be statistically significant (*p* < 0.05). The VHN of the RBCs polymerized by the LED LCU decreased from the top surface to the bottom surface, except for the Tetric Ceram and Tetric Flow RBCs placed using the bulk technique. This difference was found to be statistically significant according to the *t*-test for the standard and incremental techniques (t = 4.75 and t = 3.67, respectively; *p* < 0.01). The VHNs of the RBCs polymerized with the LED LCU on the top and bottom surfaces are given in Figure 4 and Figure 5.

According to the one-way ANOVA, the mean VHNs and standard deviations of the RBCs polymerized with the UV LCU on the top and bottom surfaces are given in Table 4.

**Figure 6:**
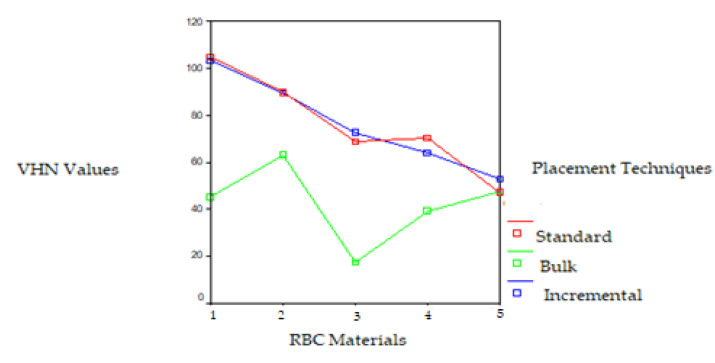
VHN of the RBCs polymerized with UV LCU on the top surface of RBC materials: 1. Filtek Z250 microhybrid, 2. Filtek P60 packable, 3. Tetric Ceram hybrid, 4. Admira ORMOCER, and 5. Tetric Flow flowable RBCs.

**Figure 7:**
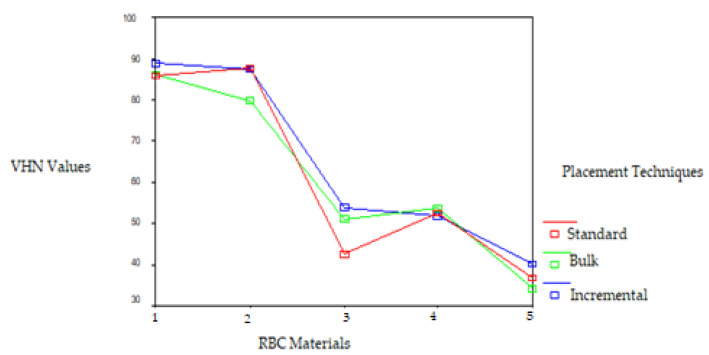
VHN values of the RBCs polymerized with UV LCU on the bottom surface of RBC materials: 1. Filtek Z250 microhybrid, 2. Filtek P60 packable, 3. Tetric Ceram hybrid, 4. Admira ORMOCER, and 5. Tetric Flow flowable RBCs.

Significant differences were observed in the VHNs of the RBCs polymerized with the UV LCU on the top and bottom surfaces. The VHNs of the RBCs polymerized by the UV LCU ranged from 104.86 to 33.87. While the Filtek Z250 RBC placed using the standard technique showed the highest VHN and the Tetric Ceram RBC placed using the bulk technique showed the lowest VHN on the top surface, the Filtek Z250 RBC placed using the incremental technique showed the highest VHN and the Tetric Flow RBC placed using the bulk technique showed the lowest VHN on the bottom surface. The VHN of the RBCs polymerized by the UV LCU decreased from the top surface to the bottom surface, except for the RBCs placed using the bulk technique. The Filtek Z250 microhybrid and packable Filtek P60 RBCs polymerized by the UV LCU exhibited higher VHNs on both surfaces than those of the other RBCs, except for the Filtek Z250 RBC placed using the bulk technique on the top surface. This difference was found to be statistically significant (*p* < 0.05). Except for the Filtek P60 RBC, all RBCs placed using the bulk technique and polymerized with the UV LCU showed lower VHNs on the top surface than those of the other placement techniques (*p* < 0.05). Very low VHNs were obtained for the Tetric Flow RBC polymerized with the UV LCU on both surfaces, except for those placed using the incremental technique. Except for the Tetric Flow RBC, all RBCs placed using the bulk technique and polymerized by the UV LCU showed higher VHNs on the bottom surface than the top surface, surprisingly. This difference was statistically significant according to the *t*-test for the standard and incremental methods (t = 4.48, t = 3.86, *p* < 0.05). The VHNs of the RBCs polymerized with the UV LCU on the top and bottom surface are given in Figure 6 and Figure 7.

## 4. Discussion

In this in vitro study, the VHNs of five different RBCs (microhybrid, packable, hybrid, ORMOCER, and flowable RBCs) placed using the standard, bulk, and incremental techniques and polymerized by LED and UV LCUs were investigated. It was observed that there were significant differences in the VHNs of the RBCs polymerized by LED and UV LCUs on the top and bottom surfaces in terms of RBC type and placement technique. Therefore, the null hypothesis that both the RBC type and placement technique would affect the hardness of the RBCs was accepted.

There are various techniques to evaluate the efficiency of polymerization of dental materials. The surface hardness test is an indicator of the degree of polymerization [28]. Microhardness enables the material to resist deformation, indentation, and scratching and predicts its resistance to abrasion and wear when used for occlusal restorations [10,34]. The Vickers and Knoop microhardness tests are the most commonly used hardness tests in dentistry [28,35]. There is a slight difference between the number values of the Knoop and Vickers microhardness tests when measured under different loads, depending on the cross-section and shape of the writing tip. While the Knoop hardness test gives more reliable results for elastic materials, the Vickers hardness test is suitable for measuring the hardness of fragile, brittle materials such as RBCs [5,35]. Since the diamond indenter used in the process has been reported to not deform over time and is suitable for measuring the hardness of composite materials, the Vickers hardness test was used in this study [34].

However, there is still no consensus on what the optimal value for Vickers hardness should be [38]. Some authors believe that for RBCs, a hardness value must exceed 50 (VHN) to be considered ideal [34,39]. In the present study, the VHNs of the RBCs polymerized by the LED LCU ranged between 110.33 and 25.16 and between 90.06 and 31.95 on the top and bottom surfaces, and the VHNs of the RBCs polymerized with the UV light source varied between 104.86 and 33.87 and between 88.87 and 34.20 on the top surface and bottom surface, respectively.

Many factors affect the surface hardness of RBCs, such as shade, resin composition, the load and size of particle fillers, the type of photoinitiator, the spectral output emitted by the LCU, light intensity, and the energy density of the LCU. The design of an LCU’s light guide, changes in the diameter of the light guide, the distance between the LCU and RBC, and curing time also affect the light distribution, power density, and, ultimately, the dentist’s ability to properly cure the RBC [1,2,10,13].

The RBCs used in this in vitro study have the same shades but different compositions. Since different color tones may change light transmittance, shade A2 of the RBC materials was used for the hardness test measurements to minimize the effect of colorants on the polymerization efficiency [40].

In this study, Filtek Z250 microhybrid and Filtek P60 packable RBCs placed using three techniques and polymerized with LED LCU showed higher VHNs on both surfaces than other RBCs. All RBCs on the top surface showed a high and acceptable VHN when placed using the standard technique and polymerized with the second-generation LED LCU. Optimal and acceptable VHNs were obtained for the Tetric Ceram hybrid RBCs, except for those placed using the bulk technique on the top surface, and for the Admira ORMOCER RBCs, except for those placed using the incremental technique on the bottom surface when polymerized with the LED LCU. However, the Tetric Flow flowable RBC exhibited very low VHNs on both surfaces, and it did not polymerize effectively with the LED LCU regardless of placement technique.

The RBCs placed using the standard and incremental techniques and polymerized by the UV LCU exhibited higher and optimal VHNs on the top surface, except for the Tetric Flow RBCs placed using the standard technique. The Filtek Z250 microhybrid and Filtek P60 packable RBCs exhibited higher VHNs on both surfaces than the other RBCs, except for the Filtek Z250 RBC placed using the bulk technique on the top surface. While optimal and acceptable VHNs were obtained for the Tetric Ceram hybrid and Admira ORMOCER RBCs placed using the standard and incremental techniques and the Tetric Flow flowable RBCs placed using the incremental technique on the top surface, optimal and acceptable VHNs were obtained for the Tetric Ceram hybrid RBCs placed using the bulk and incremental techniques and the Admira ORMOCER RBCs placed using the three techniques on the bottom surface. All RBCs placed using the standard and incremental techniques showed high VHNs on the top surface, except for the Tetric Flow RBCs placed using the standard technique. All RBCs placed using the bulk technique showed lower VHNs on the top surface than other RBCs and could not sufficiently polymerize with the UV LCU, except for the Filtek P60 RBCs. Similarly, the Tetric Flow RBCs were not sufficiently polymerized by the UV LCU on either surface, except for those placed using the incremental technique on the top surface.

In the present study, the Filtek Z250 RBCs polymerized with both LCUs showed higher VHNs than the bulk-fill and other RBCs, which agrees well with the studies of Galvão et al., Melo et al., and El Gezawi et al. [34,41,42]. The differences in VHNs can be due to differences primarily in the composition of the resin matrix, filler size, filler volume, and filler type of the RBCs [1,2]. Galvão et al. stated that the type of resin used could influence the hardness value obtained [34]. According to the data given by the manufacturers, although the organic and inorganic parts of the RBCs evaluated in this study are similar for the Filtek Z250 microhybrid and Filtek P60 bulk-fill RBCs and for the Tetric Ceram hybrid and Tetric Flow flowable RBCs, there are differences in the particle size, shape, distribution, and volume of the filler content (Table 1). The higher VHNs obtained for the Filtek Z250 and Filtek P60 RBCs might be explained by the presence of UDMA and Bis-EMA in the organic structure of these RBCs [43]. It was declared that the type of filler, filler load, and the interactions between the filler and matrix influence the surface hardness to a greater extent than the organic matrix structure [44]. In general, the higher the filler content of an RBC, the higher the surface microhardness [1]. The different technologies used to produce them may cause them to have significantly different properties and ultrastructures. The ultrastructure, size of filler particles, shape, volume/weight fraction of the filler, and chemical composition of the RBCs had an effect on the Vickers hardness [45]. Scanning electronic microscopy coupled with energy-dispersive X-ray spectroscopy (SEM-EDX) revealed that the Z250 microhybrid RBC contains spherical, regular, and dense fillers of aluminum, silicon, zirconium, barium, and fluoride [2]. The inorganic filler contents such as silica, glass, quartz, ceramic, metal, and prepolymerized particles of various shapes and sizes can improve the mechanical properties of RBCs and achieve suitable properties for various clinical applications, such as a low shrinkage volume and a low level of stress, as well as desirable viscosity, color, and biocompatibility [5]. Higher fillers consisting of silica and zirconia particles may be the reason why Filtek Z 250 and Filtek P60 RBCs have high VHNs compared to the other RBCs tested [1,45]. The P60 bulk-fill RBCs polymerized with both LCUs showed higher VHNs than the other RBCs, consistent with the findings of the studies by Saati et al. and Garoushi et al. [46,47]. It has been declared that more translucent RBCs, such as bulk-fill RBCs, allow for more light transmission from the LCU through the layers and the entire RBC thickness, and this results in a higher VHN [46].

The ORMOCER-based materials showed intermediated hardness values when compared to the conventional and packable RBCs. Moreover, the ORMOCER contains barium glass fillers, which have a lower hardness value than the zirconia fillers used in the Filtek Z50 and Filtek P60 RBCs. According to the data given by the manufacturers, both Admira ORMOCER and Tetric Ceram hybrid RBCs consist of the same particle size, a 0.7 μm inorganic filler. The Admira resin material is a combination of inorganic (ceramic) and organic materials, and the filler material is composed of a special glass ceramic and highly dispersed silica and incorporated into cross-linked inorganic and organic matrix networks (Table 1). It was declared that a higher concentration of Si combined with Zr could make a composite resin harder. EDS analyses showed a higher Si filler content in the ORMOCER than the hybrid RBCs. The higher inorganic filler load level and the filler–matrix interactions probably have a greater influence on the hardness of the Admira ORMOCER RBCs than the structure of the organic matrix [45]. The Tetric Ceram hybrid RBC has more inorganic filler than the Tetric Flow flowable RBC, and there are differences between the particle size, shape, distribution, and volume of the filler content (Table 1). Second-generation LED and UV LCUs did not polymerize sufficiently the Tetric Flow flowable RBC with the recommended times, even at a 2 mm depth. It is not surprising considering it has less filler content and loading (64.6% weight) [Table 1]. Flowable RBCs were introduced to simplify placement procedures, especially narrow cavities deeper than 4 mm, better adapt to internal surfaces of cavity preparations, and improve cavity seals. However, since the flowable RBCs may not withstand the occlusal forces, they should be used as liners, especially in class II restorations under the high-viscosity bulk-fill RBCs [5,48].

To imitate the clinical situation in which the first layer of a class II restoration is cured, 8 mm thick RBCs were placed into molds using the bulk technique as a single 8 mm thick layer and the incremental technique as 4 horizontal layers with a thickness of 2 mm. It was stated that the bulk-fill RBCs have optimal physico-mechanical properties and provide a proper margin seal and internal fit. Three bulk-fill placement techniques are available. In the bulk traditional (BT) method, firstly, a high-viscosity bulk-fill RBC is placed at the proximal wall in order to transform class II into class I, then the residual cavity is filled using one single increment of a high-viscosity bulk-fill RBC. In the Bulk&Go (BG) method, the whole cavity is filled using only one single increment of a high-viscosity bulk-fill RBC. In the Bulk&Flow (BF, snowplow technique) method, firstly, a thin layer of a flowable uncured bulk-fill RBC is placed as a cavity bottom liner, then the rest of the cavity is filled using one single increment of a high-viscosity bulk-fill RBC, and both RBCs are polymerized simultaneously. μ-CT and SEM analyses indicated that the BG and BF groups showed similar results in terms of external margins and internal fit, which allows them to better adapt to the gingival wall, consequently reducing the risk of the occurrence of secondary caries. However, the BG method also simplifies class II restorations without transforming them into class I restorations, thus achieving a successful result. Therefore, the BG technique was chosen for placing the RBCs with the bulk-fill technique in this study [48]. Abed et al. (2015) found that a bulk-fill RBC placed using the incremental technique showed a higher VHN than a bulk-fill RBC placed using the bulk technique [7]. This result agrees with the study of a Filtek P60 bulk-fill RBC placed using the incremental method, which exhibited higher VHNs than bulk-fill RBCs placed using the bulk technique and polymerized with UV LCU on the top and bottom surfaces. This could be related to the greater total energy delivered to the incrementally placed RBCs [7]. The Filtek P60 bulk-fill RBCs placed using the bulk technique and polymerized with an LED LCU exhibited similar VHNs on the top and bottom surfaces.

Since RBCs contain co-initiators that need shorter wavelengths, it is important to know the type and concentrations of photoinitiators in the RBC, especially if they are polymerized with second-generation LED LCUs [24,28,29]. Therefore, the light absorption spectrum of the photoinitiator should fully or partly overlap the radiation spectrum of the dental LCU used for photopolymerization [3]. The penetration depth is also influenced by the concentration of the photoinitiator. The optimum concentration of the photoinitiator to achieve a minimum cure time depends on the type of initiator and the sample thickness. It was found that the optimum concentration decreases slightly with increasing thickness. Optimum concentrations of the photoinitiator were found to be in the range between 0.2% and 0.4% of the weight of the RBC [36]. The manufacturers stated that the Filtek P60 packable, Filtek Z250 microhybrid, and Admira ORMOCER RBCs used in this study contain CQ as a photoinitiator; however, it is unknown whether the Tetric Ceram hybrid and Tetric Flow flowable RBCs contain CQ or co-initiators, in addition to CQ. In this study, the reason for the decrease in the polymerization efficiency of the Tetric Flow flowable RBCs placed using the three techniques and polymerized by an LED LCU may be that the Tetric Flow RBCs contain co-initiators.

It was stated that the top surface hardness of the RBCs was not dependent on the light intensity or exposure time [49]. The top surface is not an adequate clinical indicator of an adequately polymerized RBC restoration, as even a weak light source can produce a well-cured surface that hides inadequate or unpolymerized resin in deeper parts of the cavity. The top surface is not a sufficient clinical indicator of an adequately polymerized RBC [7,30]. In the study, all RBCs placed using the three techniques were polymerized effectively by an LED LCU at a 229.153 mW/cm^2^ light intensity on the top surface, except for the Tetric Ceram and Tetric Flow RBCs placed using the bulk technique. All RBCs placed using the standard and incremental techniques and the Filtek P60 RBCs placed using the bulk technique were polymerized effectively by a UV LCU at a 26.106 mW/cm^2^ light intensity on the top surface.

The hardness of the bottom surface should also be close to the hardness of the top surface. However, the distance between the light tip and the bottom of the RBC increment applied increases with increasing cavity depth. It has been stated that a significant decrease in the surface microhardness of the first applied increment RBC layer in class II cavities, which is farthest from the LCU tip [1]. The light intensity is decreased due to the absorption and dispersion by inorganic fillers and the resin matrix. While larger particles and the increased filler content are the least affected by light scattering, smaller particles emit and scatter light more than larger particles [6,7,34]. This decrease leads to a decline in VHNs from the top surface to the bottom surface, which can cause improper curing on the bottom surface [9,34]. In the study, the VHN of the RBCs placed using the three techniques and polymerized by the LED LCU decreased from the top surface to the bottom surface, except for the Tetric Ceram and Tetric Flow RBCs placed using the bulk technique. This finding is compatible with the results of other studies [26,27]. The VHN of the RBCs polymerized by the UV LCU decreased from the top surface to the bottom surface, except for the Tetric Ceram and Admira RBCs placed using the bulk technique in the study, which is partly compatible with Cook (1980) and Ruyter and Oysaed (1982) [15,16]. An advantage of UV irradiation is its efficiency in curing the monomer with a high level of conversion in the surface layers due to its ability to prevent oxygen-inhibited layer formation on the surface [15,17]. Cook (1980) reported that UV wavelengths have limited penetration into RBCs as they become consumed in their top layer [16]. Ruyter and Oysaed demonstrated that the conversion at different depths and the maximum curing depth are dependent on the composition of the RBC material, light source, and exposure time. They found that maximum scattering occurs in RBCs activated by ultraviolet light when the effective size of the particles is approximately half the wavelength of the activating light [15]. The reduced amount of light, as well as the limited penetration of the UV wavelengths, may lead to inadequate polymerization in the deepest regions of the restorations. This difference can also be explained by the overlap of the emission spectrum of LCUs and emission absorption of RBCs, as mentioned before. Because RBCs filter shorter light wavelengths more than longer ones, the shorter wavelengths delivered by a UV LCU may not reach the bottom of these RBCs [26]. However, Cook and Ruyter and Oysaed studied earlier RBCs containing a benzoin methyl ether photoinitiator.

Higher VHNs were obtained using a UV LCU on the bottom surface of the RBCs placed using the bulk technique, except for the Tetric Ceram and Admira RBCs, which was a significant difference compared to the other VHNs in the study. Two possibilities may explain these results. First, the samples were prepared in one increment and were compressed so as to extrude excess material and obtain a flat surface. This could allow for the resin matrix to accumulate at the top and condense the filler at the bottom, thereby decreasing the hardness value of the top surface. Second, due to the difference in irradiance, light may have reached the bottom surface differently [30].

The LCUs and the resin–composite formulation also affect the curing time required to stabilize hardness values. The curing times recommended by the manufacturers may not be adequate to fully cure all types of RBCs to the proper depth, which all contain different photoinitiators using LCUs [31,50]. Since the light intensity decreases as it passes through the RBC, some researchers have suggested increasing the curing time to improve the penetration of light deep into the material and polymerize more monomers for effective polymerization [7,49,50,51]. In the present study, the Tetric Flow RBCs placed using the three techniques showed very low VHNs on the top and bottom surfaces and could not polymerize well by both the low-intensity LCUs with the recommended curing time according to the manufacturers, probably due to more scattering, the greater sample thickness, and the short curing time. Longer curing times might be required for polymerizing the Tetric Flow flowable RBCs to obtain optimal VHNs. Zorzin et al. (2015) suggested that increasing the curing time of bulk-fill, conventional, and flowable RBCs beyond the manufacturers’ recommendations (+10–20 s) had positive effects on the microhardness of the RBCs [51]. It also should be noted that the composite curing times recommended by the manufacturer are possible only when the curing light output is optimal. Therefore, LCUs should be monitored regularly to ensure adequate light output [52].

Variability in the applied load by the Vickers indenter also affects the obtained hardness readings. Therefore, to compare surface hardness values among studies, the applied load must be controlled [35].

Considering the results of the present study, the type of resin and placement technique had a significant effect on the VHNs of the RBCs tested. The RBC should be carefully selected if a second-generation LED LCU is used for polymerization. The photoinitiator composition and concentration of RBCs should be described in detail in the material data sheets provided by the manufacturer so that clinicians can best match the light output with the spectral needs of an RBC material [24,28,29]. It is unknown whether Tetric Ceram hybrid and Tetric Flow flowable RBCs contain co-initiators, which is a limitation of this study.

If the curing time is increased, a higher VHN could be obtained for Tetric Flow flowable RBCs polymerized with low-intensity second-generation LED and UV LCUs.

In clinical practice, especially in the posterior area, after checking occlusal contacts, the RBC is rarely left unpolished and unfinished [53,54]. However, it has been stated that polishing and finishing affect the chemical and physical properties of RBC materials depending on the type of resin. Therefore, finishing and polishing RBCs after curing are advised to obtain a smooth surface, avoid the oxygen inhibition of the surface layer, fit the occlusion, and improve their aesthetics [54]. Monterubbianesi et al. [54] reported that the polishing of high-viscosity dimethacrylate-based bulk-filled RBCs immediately and 24 h after curing resulted in an improvement in the VHN. Therefore, immediate polishing was required. However, the same polishing times did not affect the VHNs of low-viscosity methacrylate-based bulk-filled RBCs., Therefore, it was recommended to wait 24 h before polishing, since the surface was more regular when the polishing was delayed than with immediate polishing [54]. It was also stated that the surfaces must be parallel to the table of the Vickers testing device; therefore, the surfaces should be smooth and polished well for hardness measurements [35]. Since the RBC samples were polymerized on transparent Mylar strips and glass plates, very smooth and shiny surfaces were obtained; the top surface was not polished. Another limitation of the study may be that the top surface of specimens was not finished and polished. However, Park et al. (2000) stated that the hardness of the celluloid strip-finished surface of Tetric Ceram hybrid RBCs increased over time [53]. When an RBC cures in the air, oxygen diffuses into the surface, whereas it is likely to be entrapped into the RBC when cured under the matrix. Therefore, it is postulated that the radicals, which reacted with oxygen entrapped in the RBC under the celluloid strip, reacted with the unreacted monomer and polymerized over time. Considering the half-life of radicals, additional polymerization may continue until a few days after light curing. They reported that no difference in Vickers microhardness was observed between the polished and celluloid strip-finished top surfaces 6 days after the polymerization.

Since this study is in vitro, it may be different from the clinical situation. Some factors, such as saliva, temperature, food and drinks with different pH values, and harmful habits, such as bruxism, may affect the composite microhardness over time. Further studies are necessary to evaluate the mechanical and other properties of RBCs on the effective polymerization of RBCs.

## 5. Conclusions

Within the limitations of this in vitro study, the following conclusions were found:

Filtek Z250 microhybrid and Filtek P60 packable RBCs placed using three techniques and polymerized with both LCUs exhibited higher VHNs on both surfaces than other RBCs. The VHNs of Filtek Z250 microhybrid, Filtek P60 packable, and Admira ORMOSER RBCs placed using three techniques and polymerized by an LED LCU decreased from the top surface to the bottom surface.

The VHNs of all the RBCs placed using the standard and incremental techniques and polymerized with an UV LCU also decreased from the top surface to the bottom surface. All RBCs placed using the bulk technique showed the lowest VHNs compared to those of the other techniques, except for the Filtek P60 RBC on the top surface, and these RBCs could not actively polymerize with UV LCUs. Except for the Tetric Flow RBC, all RBCs placed using the bulk technique and polymerized by an UV LCU showed higher VHNs on the bottom surface than the top surface, surprisingly.

The Tetric Flow RBC did not polymerize effectively using the recommended times on either surface, regardless of the LCU used.

Based on the VHNs of the RBCs used in the study, it can be said that the polymerization of RBCs placed using the standard and incremental techniques using low-intensity second-generation LED and UV LCUs is a good alternative to conventional polymerization, and the RBCs tested can be successfully used as a restorative material that can withstand high occlusal forces in posterior and anterior teeth up to a depth of 8 mm, except for the Tetric Flow flowable RBC.

The present study is original and novel, particularly because of its focus on low-intensity second-generation LED and UV LCUs.

Most hardness studies focus on high-intensity curing for rapid polymerization, but the higher energy output of an LCU would not improve the polymerization of an RBC if the photoinitiator does not absorb the emitted light. Low-intensity curing is clinically important to reduce polymerization shrinkage stress and heat generation, which can affect the longevity of restorations. Low-intensity second-generation LED and UV LCUs can affect hardness at different depths. This study may fill a gap in the existing literature and provide new insights into polymerization efficiency under reduced-light-intensity conditions.

## Figures and Tables

**Table 1:** RBC materials used in this study and their compositions.

Material	Filtek Z250	Filtek P60	Tetric Ceram	Admira	Tetric Flow
Manufacturer	3M, ESPE, St. Paul, MN, USA	3M, ESPE, St. Paul, MN, USA	Ivoclar Vivadent, Schaan, Liechtenstein	Voco GmbH, Cuxhaven, Germany	Ivoclar Vivadent, Schaan, Liechtenstein
Type	Microhybrid	Packable, condensable, moldable	Fine-particle hybrid	High-viscosity ORMOCER	Flowable low viscosity
Resin Matrix	Bis-GMA, UDMA, Bis-EMA	Bis-GMA, UDMA, Bis-EMA	Bis-GMA, UDMA, TEGDMA	Inorganic–organic copolymers (ORMOCER), Bis-GMA, diurethane (aliphatic and aromatic) DMA, BHT, TEGDMA	Bis-GMA, UDMA, TEGDMA
Inorganic Filler Type	Zirconia/Silica	Zirconia/Silica	Barium glass, ytterbium trifluoride, barium alumino-fluoro-silicate glass, highly dispersed silicon dioxide, and spheroid mixed oxide	Barium aluminum-boro-silicate glass	Barium glass, ytterbium trifluoride, barium alumino-fluoro-silicate glass, highly dispersed silicon dioxide, and spheroid mixed oxide
Filler Loading (weight %)	82	80	79	78	64.6
Filler Content (% by volume)	60	61	60	56	39.7
Average Particle Size (μm)	0.01–3.5 μm (mean 0.6)	0.01–3.5 μm (mean 0.6)	0.04–3 μm (mean 0.7)	0.04–1.2 μm (mean 0.7)	0.04–3 μm (mean 0.7)
Co-initiator Absorption Within < 410 nm	No	no	unknown	no	Unknown
Curing Time(s) Standard Method	20 s	20 s	40 s	40 s	20 s

Data were determined according to RBC manufacturers’ information. Bis-GMA: Bisphenol A di glycidyl methacrylate; Bis-EMA: Bisphenol A polyethylene glycol diether dimethacrylate; UDMA: Urethane dimethacrylate; TEGDMA: Triethylene glycol dimethacrylate; BHT, butyl-hydroxy-toluene.

**Table 2:** Details of data of LCUs used in this study.

Light Source	Hilux Ledmax 1055 (LED LCU)	ELC-410 (UV LCU)
Manufacturer	Benlioğlu Dental Inc., Ankara, Türkiye	Eluv.Electro-Lite, Danbury, USA
Light Intensity (mW/cm^2^)	* 229.153 mW/cm^2^	* 26.106 mW/cm^2^
Lamp Output (mW/cm^2^)	1350–1500 mW/cm^2^ (2nd generation)	** UV Light Lamp Output 90 mW/cm^2^ ** Visible Light Lamp Output 600 mW/cm^2^
Emission Wavelength Range (nm)	* 418.724–512.268 nm	* 374.253–530.313 nm
Peak Wavelength (nm)	* 458.595 nm	* 465.036 nm
Light Guide Type and Diameter (mm)	** 11 mm 60° bent fiber optic	** 11 mm curvature

* Data were assessed from the emission spectra of the LCUs with the photoluminescence system. ** Data were taken from the user manual booklet prepared by the LCU manufacturers.

**Table 3:** The mean VHNs and standard deviations of RBCs polymerized with LED LCU.

LED LCU	TOP SURFACE Mean VHN ± sd (kgf/mm^2^)			BOTTOM SURFACE Mean VHN ± sd (kgf/mm^2^)		
RBCs	Standard	Bulk	Incremental	Standard	Bulk	Incremental
	$\bar{X}$ (S)	$\bar{X}$ (S)	$\bar{X}$ (S)	$\bar{X}$ (S)	$\bar{X}$ (S)	$\bar{X}$ (S)
Filtek Z 250	110.33 (1.49)	90.94 (0.89)	107.20 ^a^ (8.75)	86.26 ^Cb^ (1.98)	86.36 ^Cc^ (1.46)	90.06 (1.40)
Filtek P 60	105.13 ^A^ (2.41)	102.02 (2.02)	104.33 ^Aa^ (1.75)	83.71 ^DEb^ (2.62)	85.56 ^Dc^ (1.29)	82.52 ^E^ (2.14)
Tetric Ceram	75.0 (1.11)	46.49 (0.74)	66.66 (0.53)	56.62 (1.51)	52.08 ^d^ (1.74)	54.39 (0.55)
Admira	59.49 ^B^ (1.26)	54.64 (1.48)	58.73 ^B^ (1.53)	50.36 ^F^ (1.28)	50.30 ^Fd^ (1.45)	48.58 (0.57)
Tetric Flow	49.37 (0.50)	25.16 (0.45)	42.36 (0.35)	36.83 (0.37)	34.98 (0.36)	31.95 (0.66)

Standard deviation values are given in parentheses. According to the Tukey HSD test, the averages displayed with the same capital letter in horizontal columns are insignificant at *p* > 0.05 regarding the difference among the placement techniques. According to Scheffe and *t*-tests, the averages displayed with the same lowercase letters in vertical columns are insignificant at the *p* > 0.05 and *p* < 0.01 levels regarding the difference among RBCs.

**Table 4:** Mean VHNs and standard deviations of RBCs polymerized with UV LCU.

UV LCU	TOP SURFACE Mean VHN ± sd (kgf/mm^2^)			BOTTOM SURFACE Mean VHN ± sd (kgf/mm^2^)		
RBC	Standard	Bulk	Incremental	Standard	Bulk	Incremental
	$\bar{X}$ (S)	$\bar{X}$ (S)	$\bar{X}$ (S)	$\bar{X}$ (S)	$\bar{X}$ (S)	$\bar{X}$ (S)
Filtek Z 250	104.86 ^A^ (1.45)	45.27 ^b^ (0.92)	103.26 ^A^ (2.18)	85.76 ^Dc^ (1.28)	86.12 ^D^ (1.41)	88.87 (1.69)
Filtek P 60	89.76 ^B^ (0.85)	63.24 (0.32)	89.45 ^B^ (1.95)	87.74 ^Ec^ (1.15)	79.80 (0.69)	87.40 ^E^ (1.93)
Tetric Ceram	68.74 ^a^ (1.48)	33.87 (0.83)	72.90 (2.02)	47.42 (1.27)	51.02 ^d^ (0.94)	53.96 (1.25)
Admira	70.50 ^a^ (0.85)	39.48 (0.36)	63.99 (1.00)	52.40 ^FG^ (0.33)	53.61 ^Fd^ (0.42)	51.73 ^G^ (0.57)
Tetric Flow	47.42 ^C^ (0.42)	47.67 ^Cb^ (0.82)	52.86 (0.47)	36.76 (0.84)	34.20 (0.44)	40.16 (0.49)

Standard deviation values are given in parentheses. According to the Tukey HSD test, the averages displayed with the same capital letter in horizontal columns are insignificant at *p* > 0.05 regarding the difference among the placement techniques. According to Scheffe and *t*-tests, the averages displayed with the same lowercase letters in vertical columns are insignificant at the *p* > 0.05 and *p* < 0.01 levels regarding the difference among RBCs.

## Data Availability

The data can be requested from the corresponding authorities.
